# Risk factors for SARS-CoV-2 infection in healthcare workers following an identified nosocomial COVID-19 exposure during waves 1–3 of the pandemic in Ireland

**DOI:** 10.1017/S0950268822001595

**Published:** 2021-11-13

**Authors:** J. McGrath, C. G. McAloon, S. J. More, S. Garrett, C. Reidy, U. Geary, N. Noonan, C. Bergin

**Affiliations:** 1 Department of Genitourinary Medicine and Infectious Diseases (GUIDE), St. James's Hospital, Dublin, Ireland; 2 School of Veterinary Medicine, School of Veterinary Science Centre Belfield, University College Dublin, Dublin, Ireland; 3 Quality, Safety and Improvement Directorate, St. James's Hospital, Dublin, Ireland; 4 Occupational Health Department, St. James's Hospital, Dublin, Ireland

**Keywords:** COVID-19, epidemiology, hospital-acquired (nosocomial) infections, occupation-related infections, transmission

## Abstract

Healthcare workers (HCWs) have increased exposure and subsequent risk of infection with severe acute respiratory syndrome coronavirus-2 (SARS-CoV-2). This case-control study was conducted to investigate the contemporaneous risks associated with confirmed SARS-CoV-2 infection amongst HCWs following in-work exposure to a confirmed coronavirus disease-2019 (COVID-19) case. We assessed the influence of demographic (age, sex, nationality, high risk co-morbidities and vaccination status) and work-related factors (job role, exposure location, contact type, personal protective equipment (PPE) use) on infection risk following nosocomial SARS-CoV-2 exposure. All contact tracing records within the hospital site during waves 1–3 of the COVID-19 pandemic in Ireland were screened to identify exposure events, cases and controls. In total, 285 cases and 1526 controls were enrolled, as a result of 1811 in-work exposure events with 745 index cases. We demonstrate that male sex, Eastern European nationality, exposure location, PPE use and vaccination status all impact the likelihood of SARS-CoV-2 infection following nosocomial SARS-CoV-2 exposure. The findings draw attention to the need for continuing emphasis on PPE use and its persisting benefit in the era of COVID-19 vaccinations. We suggest that non-work-related factors may influence infection risk seen in certain ethnic groups and that infection risk in high-risk HCW roles (e.g. nursing) may be the result of repeated exposures rather than risks inherent to a single event.

## Introduction

Healthcare workers (HCW) have been a population of special interest throughout the coronavirus disease-2019 (COVID-19) pandemic as a consequence of increased exposure to the severe acute respiratory syndrome coronavirus-2 (SARS-CoV-2) and subsequent risk of infection [1]. Numerous infection prevention and control (IPC) measures have been introduced in healthcare institutions throughout the pandemic, including the use of personal protective equipment (PPE), contact tracing (CT) and early adoption of COVID-19 vaccinations. Despite these interventions, the burden that SARS-CoV-2 infection has placed on HCWs themselves and the systems in which they work has persisted, putting continuous pressure on their ability to provide patient care.

Increased risk of SARS-CoV-2 infection has been identified in HCWs of male sex [2], Black or Asian ethnicity [3, 4], nursing and healthcare assistant (HCA) job role [5, 6], while intensive care unit (ICU) settings are reported to decrease risk [7, 8]. Seroprevalence studies are often utilised when investigating HCW SARS-CoV-2 infection and are advantageous in determining cumulative risk over time, however they do not determine risks associated with a given in-work COVID-19 exposure. Furthermore, reported high infection prevalence in certain HCW groups may reflect a combination of work-related and social factors [9]. There is a need to identify the work-related features within a healthcare setting itself that increase infection risk, which may inform institutional response in further waves of the current pandemic.

This study was conducted as part of the Hospital Epidemiology, Risks, Responses and Viral Dynamics (HERD) Study, to investigate the contemporaneous risks associated with confirmed SARS-CoV-2 infection amongst HCWs following in-work exposure to a confirmed COVID-19 case. A combination of digital CT and occupational health (OH) data was utilised to identify risk factors contributing to infection. The study aimed to assess the influence of demographic (age, sex, nationality, high-risk co-morbidities including hypertension, asthma, diabetes mellitus and vaccination status) and work-related factors (job role, exposure location, contact type, PPE use) on infection risk in HCWs following nosocomial exposure to a known COVID-19 case.

## Methods

### Study design

This is a retrospective case-control study of HCWs following in-work exposure to a confirmed COVID-19 case (‘the index case’). Digital CT and OH data were used to identify factors associated with the exposure that resulted in SARS-CoV-2 infection or non-infection. For this study, an exposure event is defined as in-work contact between an index case (either a patient or staff member) and a HCW that was identified by the CT team and/or OH department. A case was defined as a HCW who developed polymerase chain reaction (PCR)-confirmed SARS-CoV-2 infection following an exposure event. Controls were HCWs who did not develop SARS-CoV-2 infection, as determined by negative PCR, following an exposure event.

### Study site

The hospital study site is located in Dublin, Ireland and is the country's largest acute academic teaching hospital with approximately 5200 full and part-time staff members. Extensive CT operations were undertaken throughout the pandemic which worked closely with the OH department through digital records, which are utilised in this study. A number of IPC measures were introduced throughout the pandemic and notably, PPE supplies during this time were maintained without significant disruption. PPE recommendations included universal mask wearing from March 2020 and the introduction of eye protection for all patient contact from May 2020. Increased PPE use was recommended for the care of known contacts with COVID-19 cases (eye protection, surgical mask, plastic apron and gloves) and suspected/confirmed COVID-19 cases (long sleeve gowns, FFP2/3 masks, eye protection).

CT/OH teams were notified electronically of all positive SARS-CoV-2 PCR results relating to staff members and inpatients. CT teams utilised a combination of staff interviews and electronic patient record interactions to determine a complete list of contacts for a given index case. Index case exposures were classified as either ‘close’ or ‘casual’ (contact status). A number of factors influenced classification, with criteria for ‘close’ including cumulative unprotected contact within a single work shift (any breach/omission of the appropriate PPE) with a known COVID-19 case, within 2 m for more than 15 min. Criteria for ‘casual’ included spending less than 15 min in face-to-face contact within 2 m of a confirmed case.

### Participant enrolment

**Fig. 1.**
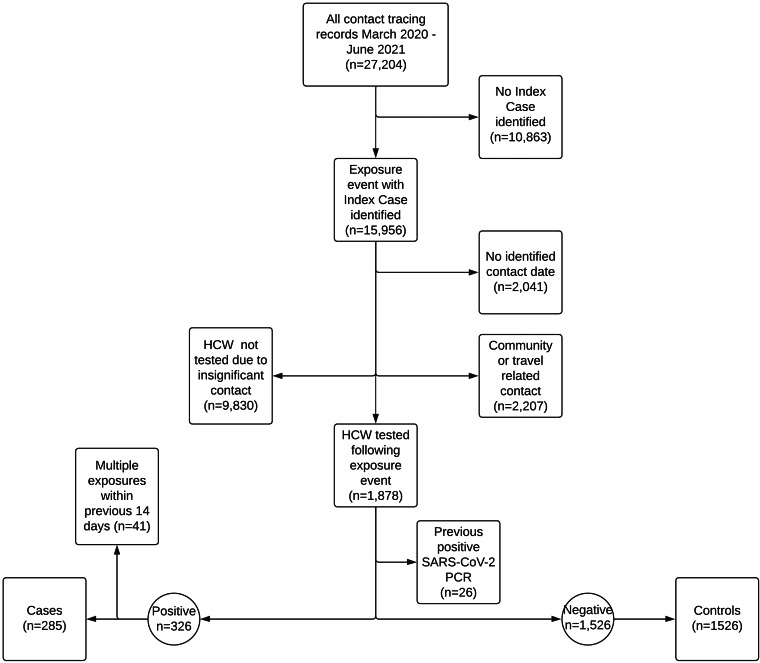
Flow diagram of participant recruitment.

All CT records within the hospital site from March 2020 to June 2021, corresponding to waves 1–3 of the COVID-19 pandemic in Ireland [10], were screened to identify exposure events, cases and controls (Fig. 1).

Inclusion criteria for cases included (1) confirmed SARS-CoV-2 via PCR, (2) an identifiable exposure event with an index case, (3) positive result identified within 14 days of exposure and (4) a known contact date. Inclusion criteria for controls included (1) a negative SARS-CoV-2 PCR result, (2) an identifiable exposure event with an index case, (3) no positive PCR result for SARS-CoV-2 within 14 days of exposure and (4) a known contact date. Cases and controls were excluded if there was evidence of exposure that arose through travel, family/household/community contact or if there was a history of a previously known positive SARS-CoV-2 PCR result prior to the study period. Cases were excluded if there were multiple confirmed exposures occurring within the preceding 14 days. HCW testing arising from blanket/ward screening, in the absence of exposure to a specific index case, were excluded. Cases and controls were not matched. All cases/controls meeting the criteria were included.

### Data collection and processing

HCW exposures were identified using CT records. Data relating to age, sex, nationality, co-morbidities (hypertension, asthma, diabetes mellitus), vaccination status, job role, exposure location, contact type (patient or staff member as index case), PPE use and dates of contact/PCR testing were derived from a combination of CT and OH databases. Data processing and participant selection is summarised in Figure 1.

### Statistical analyses

Percentages and IQR were calculated where appropriate for demographic and work-related factors. Characteristics of cases and controls were compared using the chi-square (*χ*^2^) test. Univariate logistic regression was used to determine crude odds ratios (OR) for the variables listed above and their association with SARS-CoV-2 infection following exposure to an index case. Backwards stepwise logistic regression was used to identify variables for inclusion in a final multivariable model. The *P*-values for entry into and retention in the model were 0.2 and <0.05 respectively. For each variable, adjusted odds ratios (aOR) for SARS-CoV-2 infection following exposure to an index case were calculated. Data processing was undertaken using R, version 4.1.1 (R Core Team, 2021) [11].

## Results

### Exposure events

In total, 285 cases and 1526 controls were enrolled in the study, as a result of 1811 in-work exposure events with 745 index cases between March 2020 and April 2021. This was related to wave 1–3 of the COVID-19 pandemic in Ireland. Each index case was linked to a mean of 2.5 HCW exposure events (range 1–42, median 2, IQR 1–3).

**Fig. 2.**
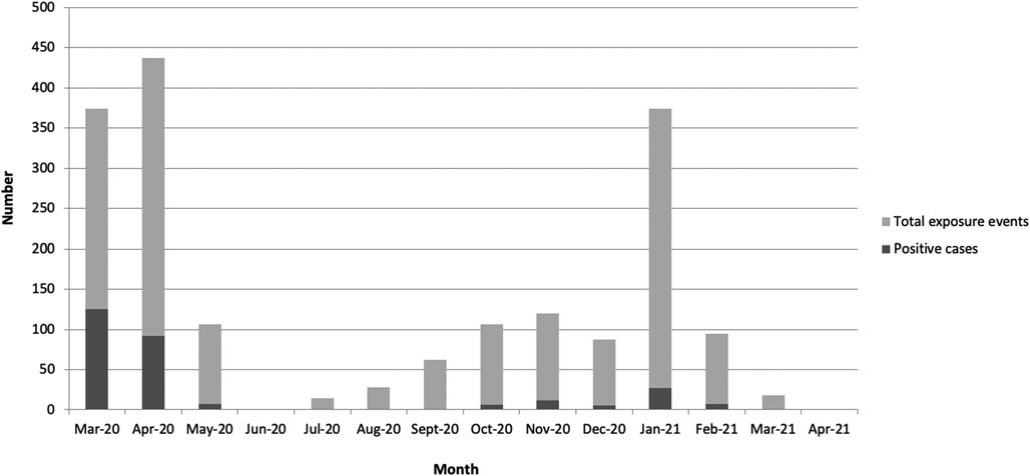
Temporal pattern of exposure events and cases during the study period.

The reported in-work exposure events occurred most frequently during March 2020 (20.7%, *n* = 374) and April 2020 (24.1%, *n* = 437). An increase in the number of exposure events was noted subsequently in January 2021 (20.7%, *n* = 374). In total, 51.4% (*n* = 931) of exposure events occurred during wave 1 (March–August 2020), 17.2% (*n* = 312) during wave 2 (August–November 2020) and 31.4% (*n* = 568) during wave 3 (November 2020–June 2021). The number of exposure events and cases over time is presented in Figure 2.

### Description of cases

Demographic and work-related factors for cases are detailed in Table 1. Of the 285 cases, 74.4% (*n* = 212) were female and 25.7% male (*n* = 73) with a median age 37 years (range 20–67, IQR 29–47 years). Distribution of cases over time is shown in Figure 2. Irish nationality was most commonly reported (55.4%, *n* = 158), followed by Indian (12.6%, *n* = 36) and Filipino (11.2%, *n* = 32). Hypertension was present in 6.7% (*n* = 19) of cases, asthma in 8.4% (*n* = 24) and diabetes mellitus (type 1 or 2) in 3.5% (*n* = 10).

**Table 1.** Demographic and work-related factors for cases and controls

	Cases (*n* = 285)	Controls (*n* = 1526)	*P*-value^a^
	*n* (%)	*n* (%)	
Sex			
Male	73 (25.7)	316 (20.7)	0.06
Female	212 (74.4)	1210 (79.3)	
Age			
18–25	34 (11.9)	209 (13.7)	0.6
26–35	102 (35.8)	531 (34.8)	
36–45	67 (23.5)	355 (23.3)	
46–55	54 (18.9)	316 (20.7)	
56–65	28 (9.8)	112 (7.3)	
>65	0	3 (0.2)	
Range (years)	20–65	20–67	
Median	37	36	
Mean	38.5	38	
IQR	29–47	29–46	
Nationality			
Irish	158 (55.4)	935 (61.3)	0.02
Indian	36 (12.6)	206 (13.5)	
Filipino	32 (11.2)	174 (11.3)	
Eastern European	13 (4.6)	30 (2)	
African	6 (2.1)	37 (2.4)	
Other	40 (14)	144 (9.4)	
Job role			
Nursing	161 (56.5)	787 (51.6)	0.08
HCA	45 (15.8)	190 (12.5)	
Medical	28 (9.8)	189 (12.4)	
General support	21 (7.4)	115 (7.6)	
Allied Health	15 (5.3)	138 (9)	
Administration	14 (4.9)	84 (5.5)	
Other	1 (0.4)	23 (1.5)	
Vaccination status			
Unvaccinated	273 (95.8)	1361 (89.2)	<0.001
First dose	12 (4.2)	104 (6.8)	
Both doses	0	61 (4)	
Exposure location			
Medical ward	97 (34)	596 (39.1)	<0.001
COVID ward	56 (19.7)	144 (9.4)	
Surgical ward	29 (10.2)	171 (11.2)	
Acute medical ward	17 (6)	87 (5.7)	
Emergency	10 (3.5)	48 (3.1)	
Outpatients	8 (2.8)	45 (3)	
Intensive care unit	7 (2.5)	75 (4.9)	
Administration	6 (2.1)	25 (1.6)	
Other	55 (19.3)	335 (22)	
Contact type			
Patient – staff wearing PPE	59 (20.7)	411 (26.9)	0.02
Patient – staff not wearing PPE	61 (21.4)	233 (15.3)	
Patient – PPE unknown	12 (4.2)	31 (2.2)	
Staff	128 (44.9)	713 (46.7)	
Other/unknown^b^	25 (8.8)	138 (9)	
Contact status			
Close	181 (63.5)	894 (58.8)	0.2
Casual	91 (31.9)	573 (37.5)	
Unknown	13 (4.6)	59 (3.9)	
Co-morbidities			
Asthma	24 (8.4)	118 (7.7)	0.5
Hypertension	19 (6.7)	76 (5.0)	
Diabetes	10 (3.5)	30 (2)	

a *P*-values determined via chi-square (*χ*^2^) test.

b Contact type ‘Other/unknown’ includes exposures with an identified index case but where patient/staff status of the index case was not known.

Nursing was the most common job role reported (56.5%, *n* = 161), followed by HCA (15.8%, *n* = 45) and medical roles (9.8%, *n* = 28). General medical wards were the most frequent site of exposure to an index case (34%, *n* = 97) followed by COVID-19 wards (19.7%, *n* = 56) and surgical wards (10.2%, *n* = 29). For 46.3% (*n* = 132) of cases, the exposure source (index case) was a patient, with PPE being worn by the HCW in 59 patient exposures (44.7%) and not worn in 61 (46.2%). PPE status was unknown in 9.1% (*n* = 12) of patient exposures. Another staff member was the index case in 44.9% (*n* = 128) of cases, with location of staff–staff exposure similar to that of staff–patient exposure (medical wards (29%) and COVID wards (20.3%) most frequently). Exposure events were considered either close (63.5%, *n* = 181) or casual (31.9%, *n* = 91), with 4.6% (*n* = 13) not classified. The majority were unvaccinated (95.8%, *n* = 273) at the time of exposure, with 4.2% (*n* = 12) having received one dose of COVID-19 vaccine, at least 7 days prior to exposure. The time interval between index case exposure and PCR-testing was median 5 days (range 2–14, IQR 3–8).

### Description of controls

Demographic and work-related factors for controls are detailed in Table 1. Within the control group (*n* = 1526), median age was 38 years (range 20–67, IQR 29–46 years) and 79.3% (*n* = 1210) were female. Irish nationality was most commonly reported (61.3%, *n* = 935) followed by Indian (13.5%, *n* = 206). Hypertension was reported in 5.0% (*n* = 76) of controls, asthma in 7.7% (*n* = 118) and diabetes mellitus (type 1 or 2) in 2% (*n* = 30).

Nursing was the most common job role (51.6%, *n* = 787) and the most frequent site of exposure to an index case was medical wards (39.1%, *n* = 596). Eighty-nine per cent (*n* = 1361) were unvaccinated at the time of exposure, with 10.8% (*n* = 165) receiving at least one vaccine dose prior to exposure. Another staff member was the most common index case exposure amongst controls (46.7%, *n* = 713). The time interval between index case exposure and PCR-testing was median 4 days (range 1–14, IQR 3–6), though many controls were tested multiple times following a given exposure.

### Risk factor analysis

Candidate variables meeting the multivariable model entry criteria included sex, age, nationality, job role, exposure location, vaccine status, contact type, contact status, presence of diabetes mellitus and wave of pandemic. Variables remaining in the final multivariable model are detailed below and in Table 2.

**Table 2.** Results of final multivariable model demonstrating risk factors for SARS-CoV-2 positivity following in-hospital exposure to a known COVID-19 index case

	*N* (%)	Crude OR (95% CI)	Adjusted OR (95% CI)	*P*-value^a^
Sex				
Female (Ref)	1422 (78.5)			
Male	**389** (**21.5)**	**1.31** (**0.97–1.77)**	**1.42** (**1.04–1.95)**	**0**.**031**
Nationality				
Irish (Ref)	1093 (60.3)			
Indian	242 (13.4)	1.03 (0.8–1.53)	0.88 (0.55–1.35)	0.561
Filipino	206 (11.4)	1.1 (0.72–1.66)	0.85 (0.55–1.32)	0.473
African	43 (2.4)	0.94 (0.39–2.26)	0.78 (0.31–1.95)	0.588
Eastern European	**43** (**2.4)**	**2.58** (**1.33–5.05)**	**3.33** (**1.6–6.93)**	**0**.**001**
Other European	40 (2.2)	1.99 (0.95–4.14)	1.56 (0.7–3.48)	0.281
Asian/Australia Pacific	29 (1.6)	0.69 (0.22–2.3)	0.78 (0.22–2.74)	0.694
North American	11 (0.6)	0 (0, Inf)	0 (0, Inf)	0.974
Latin American/Caribbean	1 (0.05)	0 (0, Inf)	0 (0, Inf)	0.993
Other/unknown	103 (5.7)	2.06 (1.29–3.3)	1.38 (0.83–2.28)	0.21
Exposure location				
Medical ward (Ref)	693 (38.3)			
Intensive care unit	**82** (**4.5)**	**0.58** (**0.26–1.29)**	**0.35** (**0.15–0.79)**	**0**.**012**
Acute medical ward	104 (5.7)	1.2 (0.68–2.1)	0.81 (0.44–1.46)	0.476
Administration/clerical	31 (1.7)	1.49 (0.6–3.71)	2.13 (0.79–5.77)	0.137
Allied health	20 (1.1)	1.1 (0.32–3.81)	1.48 (0.4–5.5)	0.562
COVID-19 ward	**200** (**11.0)**	**2.4** (**1.74–3.52)**	**2.07** (**1.39–3.08)**	**<0.001**
Emergency department	58 (3.2)	1.27 (0.61–2.58)	1.29 (0.6–2.78)	0.512
Laboratory	39 (2.2)	0.71 (0.25–2.04)	1.43 (0.46–4.39)	0.534
Oncology/haematology ward	49 (2.7)	0.26 (0.06–1.1)	0.59 (0.14–2.54)	0.476
Other^b^	157 (8.7)	1.59 (1.02–2.48)	1.75 (1.08–2.81)	0.022
Outpatients department	53 (2.9)	1.1 (0.5–2.41)	1.2 (0.52–2.75)	0.671
Pharmacy	5 (0.3)	1.55 (0.17–14.04)	1.79 (0.17–18.45)	0.627
Psychiatric ward	20 (1.1)	1.55 (0.51–4.74)	2.9 (0.88–9.63)	0.081
Radiology department	17 (0.9)	0.39 (0.05–2.96)	0.52 (0.06–4.19)	0.54
Support	56 (3.1)	0.74 (0.31–1.78)	1.17 (0.46–2.96)	0.735
Surgical ward	200 (11.0)	1.04 (0.64–1.65)	1.3 (0.8–2.09)	0.286
Theatre	26 (1.4)	0.51 (0.17–2.3)	0.77 (0.17–3.49)	0.736
Contact type				
Patient – staff not wearing PPE (ref)	294 (16.2)			
Patient – PPE unknown	43 (2.4)	1.36 (0.65–2.79)	1.11 (0.56–2.5)	0.817
Patient – staff wearing PPE	**470** (**26.0)**	**0.56** (**0.38–0.82)**	**0.59** (**0.39–0.9)**	**0**.**01**
Staff	841 (46.4)	0.69 (0.48–0.97)	0.88 (0.6–1.28)	0.053
Other/unknown	163 (9.0)	0.7 (0.42–1.15)	0.66 (0.35–1.08)	0.094
Vaccine status				
Unvaccinated (Ref)	1634 (90.2)			
At least one dose received	**177** (**9.8)**	**0.37** (**0.2–0.69)**	**0.4** (**0.23–0.77)**	**0**.**006**
Timepoint in pandemic				
Wave 1 (Ref)	931 (51.4)			
Wave 2	**312** (**17.2)**	**0.21** (**0.13–0.34)**	**0.18** (**0.11–0.3)**	**<0.001**
Wave 3	**568** (**31.4)**	**0.24** (**0.18–0.34)**	**0.21** (**0.14–0.34)**	**<0.001**

The variables highlighted as bold in this table are those variables that had significant findings on multivariable analysis.

a *P*-values relate to adjusted odd ratios (OR).

b Exposure location ‘Other’ includes miscellaneous hospital locations such as tutorial rooms, doctor's residence, chapel and non-administrative offices.

The timepoint in the pandemic in which the exposure occurred was significantly associated with likelihood of infection following exposure. March 2020 and April 2020 were the months of highest risk, with OR of 6.31 (95% CI 2.84–14.03, *P* < 0.001) and 3.35 (95% CI 1.57–7.49, *P* = 0.003) respectively. Risk reduced significantly as time progressed in the pandemic with exposures during waves 2/3 being significantly less likely than wave 1 to result in infection following a nosocomial exposure with aOR 0.18 (95% CI 0.11–0.3, *P* < 0.001) and aOR 0.21 (95% CI 0.14–0.34, *P* < 0.001) for waves 2/3 respectively (Table 2).

A number of factors were associated with increased risk of SARS-CoV-2 infection following exposure to a COVID-19 index case in the final multivariable model, including male sex, nationality, exposure location, contact type, vaccination status and timepoint in the pandemic. After accounting for all of the other variables in the model, the following were at increased risk of infection following exposure: males (aOR 1.42, 95% CI 1.04–1.95, *P* = 0.031; females as referent), Eastern European nationalities (aOR 3.33, 95% CI 1.61–6.93, *P* = 0.001; Irish national as referent) and COVID wards (aOR 2.07, 95% CI 1.39–3.08, *P* < 0.001; medical wards as referent). The following were each at decreased risk of infection following exposure: staff use of PPE (aOR 0.59, 95% CI 0.39–0.9, *P* = 0.01; no use of PPE as referent), ICUs (aOR 0.35, 95% CI 0.15–0.79, *P* = 0.012; medical wards as referent) and receipt of at least one vaccine dose (aOR 0.4, 95% CI 0.23–0.77, *P* = 0.006; unvaccinated as referent). Full risk factor analyses are detailed in Table 2.

## Discussion

We investigated risk factors associated with HCW SARS-CoV-2 infection following in-work exposure to an index case during waves 1–3 of the COVID-19 pandemic. Data collected by CT/OH teams provide contemporaneous assessment of risks and relevant characteristics of the exposure at the time of contact. The inclusion and exclusion criteria utilised aim to exclude exposures where a contact pathway is not clear or where multiple contacts within a short period of time may decouple the result from the exposure. The effect of possible post-infection immunity is mitigated by excluding from study any HCW with a history of a previously positive SARS-CoV-2 PCR result prior to the study period. The exclusion of exposures where the cited source is community-based ensures that interactions studied occur within the hospital setting.

Evidence for many of the known risk factors in HCW SARS-CoV-2 infection is derived from seroprevalence studies and while that study design is advantageous given the high rate of asymptomatic infection (estimated to range between 26% and 40% [12–14]), such investigations represent cumulative risk over time rather than assessment of a single exposure. Furthermore, the consequence of community-based factors cannot be delineated from work-related factors in such studies. The results presented here, using contemporaneous data, make work-related risk factors more readily assessable and identify risks that are consistent throughout the course of the pandemic.

### Variation in risk over time

Risk of HCW nosocomial SARS-CoV-2 infection was highest in the early stages of the pandemic (wave 1) as case definitions, isolation protocols and PPE guidance were rapidly evolving. Case numbers of confirmed SARS-CoV-2 infections in Ireland were significantly increased during waves 2 and 3 [10], reaching a peak weekly incidence of 1196.9 per 100 000 population in Dublin county during week 1 2021 [15]. Our findings demonstrate significantly reduced odds of nosocomial infection during this period (wave 2 aOR 0.18 and wave 3 aOR 0.21, respectively), likely reflecting the increased incidence and importance of community SARS-CoV-2 transmission over time, with concurrent consolidation of hospital protocols aiming to reduce in-hospital transmission.

### Sex

Our findings demonstrate an increased risk of SARS-CoV-2 infection following exposure to an index case in male HCWs with an aOR 1.42 (95% CI 1.04–1.95, *P* = 0.031). Male sex has been identified as a risk factor in HCWs for SARS-CoV-2 seropositivity in a number of studies [2, 16, 17] with quoted odds ratios ranging from 1.39 to 3.21. The Prevalence of COVID-19 in Irish Healthcare Workers Study, an independent multicentre seroprevalence study of anti-SARS-CoV-2 antibodies in HCWs in Ireland, which included the current study site, found an increased risk of SARS-CoV-2 seropositivity in male HCWs with an aRR 1.2 (95% CI 1.0–1.4, *P* = 0.016) [13]. Studies have demonstrated heterogeneous immune responses to SARS-CoV-2 infection in men and women, including delayed peaks in anti-SARS-CoV-2 immunoglobulins in men [18, 19], however these findings generally are inconclusive and lack statistical analysis to relate these findings to infectivity or disease severity [20].

### Nationality

HCWs of Eastern European nationality were found to be at higher risk of infection following exposure in comparison to HCWs of Irish nationality (aOR 3.33, 95% CI 1.61–6.93, *P* = 0.001). A number of factors may contribute to this finding. From December 2020 to September 2021, COVID-19 vaccination uptake amongst eligible men and women was significantly lower in Eastern European nationals working in Ireland (39% and 49% uptake respectively) in comparison to their Irish counterparts (89% and 92% uptake respectively) [21]. Given the protective effect COVID-19 vaccination plays in protection from SARS-CoV-2 infection [22–24], including findings presented below, this may be a factor in the increased rates of infection.

Many studies have examined ethnicity/nationality differences in SARS-CoV-2 seroprevalence and infection outcomes in HCW [1, 3, 8, 25–28]. Black [3–5, 29], Asian [4, 5] and Hispanic [5] ethnicity have been identified as high risk for SARS-CoV-2 seroprevalence and poorer infection outcomes. Delineation between nationality and ethnicity was not possible in our dataset due to local recording methodology. However, it is noted in our findings that HCWs of African (aOR 0.78, 95% CI 0.31–1.95, *P* = 0.588), Indian (aOR 0.88, 95% CI 0.55–1.35, *P* = 0.561), Filipino (aOR 0.85, 95% CI 0.55–1.32, *P* = 0.473) and other Asian/Australia Pacific (aOR 0.78, 95% CI 0.22–2.74, *P* = 0.694) nationalities did not demonstrate an increased risk of SARS-CoV-2 infection following exposure to an index case, in comparison to their Irish co-workers. Determining to what extent work-related and socioeconomic factors contribute to increased infection amongst certain ethnic groups has been noted to be challenging [9]. Our findings support the suggestion that non-work-related factors play a role in these differential rates, as it appears that in a given work-related exposure, African or Asian nationality did not confer increased risk of infection in comparison to Irish, predominantly Caucasian, colleagues.

### Job role

Variability in SARS-CoV-2 infection by job role has been demonstrated in a number of studies, with nursing staff and HCAs [5, 6, 13, 14, 30] often cited as being at increased risk of infection. In our findings, no individual job role was determined to have consistently increased risk of infection following a given in-work COVID-19 exposure. This is likely due to a combination of factors. In the study site, PPE supplies were maintained throughout the pandemic and a high level of staff compliance was observed. Given the protective role PPE plays in prevention of infection (see below), in a given interaction, a nursing or HCA HCW is protected to the same extent as other roles. The increased risk observed in other studies may be a reflection of cumulative risk, with certain HCW roles subject to an increased number of exposures over time, increasing the likelihood of infection. Furthermore, nursing staff constitute a significant proportion of many hospitals' total staff numbers (40.1% in the current study site) and this may result in over representation in seroprevalence studies, influencing interpretation of risk [13, 14].

### Location of exposure

Dedicated COVID-19 wards were found to be significantly associated with increased likelihood of infection following exposure to an index case (aOR 2.07, 95% CI 1.39–3.08, *P* < 0.001), in comparison to medical wards. Frequent contact with known COVID-19 cases increases risk of infection [13, 14, 31] and notably, it has been demonstrated that despite adequate access to PPE, this risk persists with daily exposure to COVID-19 cases [1]. The cause is likely multi-factorial including high staff turnover in COVID-19 wards leading to increased PPE failures, increased duration of interaction with non-sedated patients and an increased number of high-risk interactions per staff member. Exposure location for cases was similar in patient–staff and staff–staff interactions, which may reflect HCWs spending prolonged periods in close proximity to SARS-CoV-2-infected colleagues during ward duties.

HCW exposure to a COVID-19 index case within the ICU was associated with a 65% decreased odds of infection as compared to exposures occurring on general medical wards (aOR 0.35, 95% CI 0.15–0.79, *P* = 0.012). This finding is consistent with those of a number of other studies which demonstrate reduced SARS-CoV-2 infection risk in high dependency/intensive care settings [3, 7, 8, 25, 28], with risk reductions of up to 56% being demonstrated [3]. The protective effect in these settings despite engagement in high-risk aerosol-producing procedures may be a result of strict compliance with IPC measures and a relatively reduced patient turnover compared to other areas of the hospital.

### Preventative strategies: PPE use and COVID-19 vaccination

The protective effect mediated by PPE use varies widely in the literature, with reductions in SARS-CoV-2 infection rates from 26% to 100% reported [1, 32, 33], although this is heavily influenced by the level of PPE used and availability of supplies during the pandemic [33, 34]. In our study hospital site, PPE supplies have been maintained throughout the pandemic, with universal masking for all HCW recommended from March 2020. Our findings demonstrate that following exposure to a COVID-19 patient, use of PPE (compared with not) reduced the odds of HCW infection by 41%. The protective effect of PPE remained significant after adjusting for other covariates, including COVID-19 vaccination and location of exposure (aOR 0.59, 95% CI 0.39–0.9, *P* = 0.01).

Receipt of at least one dose of COVID-19 vaccine was associated with a reduced odds of infection by 60% (aOR 0.4, 95% CI 0.23–0.77, *P* = 0.006). The BNT162b2 vaccine was most commonly received during the study period, in keeping with the national rollout of COVID-19 vaccines in Ireland from December 2020 [35]. This study is not designed to assess vaccine efficacy and many exposures occurred prior to their availability. As a result, the protective effect demonstrated is lower than that seen in clinical efficacy studies for BNT162b2 (95%) [22] and ChAdOx1 (74%) [23]. Despite only capturing initial rollout effects in our data, vaccination remained protective, demonstrating a key role in the protection of HCWs against nosocomial infection.

### Co-morbidities

It is well-recognised that age, cardiovascular disease and diabetes mellitus are risk factors for poor outcomes in COVID-19 [36–38]. Large cohort studies have estimated prevalence of up to 30% for diabetes and hypertension in COVID-19 patients [39, 40] and evidence suggests that asthma patients may be more susceptible to contracting SARS-CoV-2 [41]. In our findings, a history of diabetes mellitus (OR 1.83, 95% CI 0.88–3.78, *P* = 0.1), hypertension (OR 1.37, 95% CI 0.82–2.31, *P* = 0.23) or asthma (OR 1.1, 95% CI 0.68–1.77, *P* = 0.7) was not significantly associated with infection; however, a trend towards significance was demonstrated with OR greater than one in each case. A larger dataset may have sufficient statistical power to identify an association with increased odds of SARS-CoV-2 acquisition for these co-morbidities. Additionally, the median age within the study cohort is 36 years and therefore the younger workforce described may underestimate the influence of these comorbidities on SARS-CoV-2 infection among HCWs.

### Limitations

The authors acknowledge a number of limitations with the current study. While inclusion/exclusion criteria applied aim to minimise unknown exposures, in the absence of concurrent whole genome sequencing (WGS) of paired SARS-CoV-2 isolates between index case and HCW, it is not possible to comprehensively exclude the possibility of an undocumented SARS-CoV-2 exposure resulting in the positive test reported. Furthermore, the retrospective nature of this study means that investigation is limited to those exposure events that were identified and pursued by the CT/OH teams and resulted in testing of the HCW. While testing was advocated for close contacts throughout the pandemic and subsequently extended to casual contacts in the hospital site, the evolving nature of testing protocols may have had an impact on case ascertainment. The findings above identify a number of factors increasing risk of SARS-CoV-2 infection following exposure, however determining specific causal links, e.g. between Eastern European nationality (of whom a small sample size of *n* = 43 was available) and vaccination rates, is outside the scope of this study. Data in relation to HCW smoking status were not consistently available. It is noted that some staff–staff ward exposures may have occurred in on-ward break rooms, however due to recording methodology this was not delineated.

## Conclusion

Within a hospital setting, we demonstrate that male sex, Eastern European nationality, exposure location, PPE use and vaccination status all impacted the likelihood of SARS-CoV-2 infection following nosocomial exposure to a known COVID-19 case during waves 1–3 of the pandemic. The findings draw attention to the need for continuing emphasis on PPE use and its persisting benefit in the era of COVID-19 vaccinations. We suggest that non-work-related factors may influence infection risk seen in certain ethnic groups and that infection risk in high-risk HCW roles (e.g. nursing) may be the result of repeated exposures rather than risks inherent to a single event. We recommend that future studies combine epidemiological and WGS data to allow a more complete assessment of outcomes after excluding, or otherwise controlling for, undocumented exposures.

## Data Availability

The authors may be contacted for discussion/access to anonymised datasets.
